# Beneficial and harmful effects of antidepressants versus placebo, ‘active placebo’, or no intervention for adults with major depressive disorder: a protocol for a systematic review of published and unpublished data with meta-analyses and trial sequential analyses

**DOI:** 10.1186/s13643-021-01705-6

**Published:** 2021-05-25

**Authors:** Sophie Juul, Faiza Siddiqui, Marija Barbateskovic, Caroline Kamp Jørgensen, Michael Pascal Hengartner, Irving Kirsch, Christian Gluud, Janus Christian Jakobsen

**Affiliations:** 1 Stolpegaard Psychotherapy Centre, Mental Health Services in the Capital Region of Denmark, Stolpegaardsvej 28, 2820 Gentofte, Denmark; 2 Department of Psychology, University of Copenhagen, Østre Farimagsgade 2A, København K, 1353 Copenhagen, Denmark; 3 Copenhagen Trial Unit, Centre for Clinical Intervention Research, The Capital Region, Copenhagen University Hospital -- Rigshospitalet, Blegdamsvej 9, 2100 Copenhagen, Denmark; 4 Department of Applied Psychology, Zurich University of Applied Sciences, Pfingstweidstrasse 96, 8005 Zurich, Switzerland; 5 Program in Placebo Studies, Harvard Medical School, 330 Brookline Avenue, Boston, Massachusetts 02215 USA; 6 Department of Regional Health Research, Faculty of Health Sciences, University of Southern Denmark, J.B. Winsløws Vej 19, 3, 5000 Odense C, Denmark

## Abstract

**Background:**

Major depressive disorder is one of the most common, burdensome, and costly psychiatric disorders worldwide. Antidepressants are frequently used to treat major depressive disorder. It has been shown repeatedly that antidepressants seem to reduce depressive symptoms with a statistically significant effect, but the clinical importance of the effect sizes seems questionable. Both beneficial and harmful effects of antidepressants have not previously been sufficiently assessed. The main objective of this review will be to evaluate the beneficial and harmful effects of antidepressants versus placebo, ‘active placebo’, or no intervention for adults with major depressive disorder.

**Methods/design:**

A systematic review with meta-analysis will be reported as recommended by Preferred Reporting Items for Systematic Reviews and Meta-Analysis (PRISMA), bias will be assessed with the Cochrane Risk of Bias tool-version 2 (ROB2), our eight-step procedure will be used to assess if the thresholds for clinical significance are crossed, Trial Sequential Analysis will be conducted to control for random errors, and the certainty of the evidence will be assessed with the Grading of Recommendations Assessment, Development and Evaluation (GRADE) approach. To identify relevant trials, we will search both for published and unpublished trials in major medical databases from their inception to the present. Clinical study reports will be obtained from regulatory authorities and pharmaceutical companies. Two review authors will independently screen the results of the literature searches, extract data, and perform risk of bias assessment. We will include any published or unpublished randomised clinical trial comparing one or more antidepressants with placebo, ‘active placebo’, or no intervention for adults with major depressive disorder. The following active agents will be included: agomelatine, amineptine, amitriptyline, bupropion, butriptyline, cianopramine, citalopram, clomipramine, dapoxetine, demexiptiline, desipramine, desvenlafaxine, dibenzepin, dosulepin, dothiepin, doxepin, duloxetine, escitalopram, fluoxetine, fluvoxamine, imipramine, iprindole, levomilnacipran, lofepramine, maprotiline, melitracen, metapramine, milnacipran, mirtazapine, nefazodone, nortriptyline, noxiptiline, opipramol, paroxetine, protriptyline, quinupramine, reboxetine, sertraline, trazodone, tianeptine, trimipramine, venlafaxine, vilazodone, and vortioxetine. Primary outcomes will be depressive symptoms, serious adverse events, and quality of life. Secondary outcomes will be suicide or suicide attempt, suicidal ideation, and non-serious adverse events.

**Discussion:**

As antidepressants are commonly used to treat major depressive disorder in adults, a systematic review evaluating their beneficial and harmful effects is urgently needed. This review will inform best practice in treatment and clinical research of this highly prevalent and burdensome disorder.

**Systematic review registration:**

PROSPERO CRD42020220279

**Supplementary Information:**

The online version contains supplementary material available at 10.1186/s13643-021-01705-6.

## Background

### Description of participants

Major depressive disorder is estimated by the World Health Organization (WHO) to affect more than 264 million people globally, making the disorder one of the leading causes of disability worldwide [1, 2]. The estimated lifetime prevalence of major depressive disorder is between 10 and 20% [3, 4]. In 2010, the annual economic burden in the USA alone was estimated to exceed 210 billion US dollars including both direct medical costs and indirect costs related to work ability and comorbidities [5]. Major depressive disorder is characterised by depressed mood and loss of interest or pleasure resulting in significant psychological distress and functional impairment [6, 7]. Furthermore, risks of suicides and suicide attempts significantly increases during major depressive episodes [8, 9]. Together, these findings emphasise the need for efficacious and cost-effective treatments for this burdensome and highly prevalent psychiatric disorder, especially treatments where benefits outweigh harms.

### Description of interventions

Pharmacotherapy is widely used in the treatment of major depressive disorder, particularly in the Western world, but also in several other countries [10, 11]. Data published in 2017 from the National Health and Nutrition Examination Survey showed that during 2011–2014, about one in eight people aged 12 and above in the USA reported taking antidepressants during the previous month [12]. The use of antidepressants has increased nearly 65% over a 15-year time frame [12] and more than 60% of people in the USA taking antidepressants have been taking them for more than two years [12]. Today, antidepressants for major depressive disorder, either alone or in combination with psychotherapy, are recommended by the UK National Institute for Health and Care Excellence (NICE) and the American Psychiatric Association, as well as different national clinical guidelines [13–19].

Several different antidepressants exist. Before the late 1980s, pharmacological treatment was limited to tricyclic antidepressants (TCAs) and monoamine oxidase inhibitors (MAOIs). TCAs and MAOIs are now commonly referred to as first-generation antidepressants. Now, second-generation antidepressants comprise most antidepressant prescriptions. These drugs include selective serotonin reuptake inhibitors (SSRIs), serotonin and norepinephrine reuptake inhibitors (SNRIs), and other drugs with related mechanisms of action that selectively target neurotransmitters. For an overview of the different types of antidepressants, please see Table 1 [10, 20, 21].

**Table 1:** Types of antidepressants

Classes of antidepressants	Examples
Tricyclic antidepressants (TCA)	Amineptine, amitriptyline, amoxapine, butriptyline, cianopramine, clomipramine, desipramine, demexiptiline, dibenzepin, dosulepin, dothiepin, doxepin, imipramine, iprindole, lofepramine, maprotiline, melitracen, metapramine, nortriptyline, noxiptiline, opipramol, protriptyline, tianeptine, trimipramine, and quinupramine.
Tetracyclic antidepressants (TeCAs)	Trazodone
Monoamine oxidase inhibitors (MOI)	Isocarboxazid and phenelzine
Selective serotonin reuptake inhibitors (SSRI)	Citalopram, dapoxetine, escitalopram, fluoxetine, fluvoxamine, paroxetine, and sertraline
Serotonin-norepinephrine reuptake inhibitors (SNRI)	Desvenlafaxine, duloxetine, levomilnacipran, milnacipran, and venlafaxine
Selective noradrenaline reuptake inhibitors (NRI)	Reboxetine
Atypical antidepressants	Agomelatine, bupropion, mirtazapine, and nefazodone, vilazodone, and vortioxetine

### How the interventions might work

Antidepressants aim to increase the availability of specific neurotransmitters that are sought to play a role in the development of major depressive disorder, most commonly serotonin, noradrenaline, and dopamine. The various antidepressants target different neurotransmitters [22]. For example, selective serotonin reuptake inhibitors (e.g., citalopram and fluoxetine) specifically block the reuptake of serotonin, while selective noradrenaline reuptake inhibitors (e.g. reboxetine) specifically block the reuptake of noradrenaline. Some antidepressants simultaneously block the reuptake of both serotonin and noradrenaline (e.g. duloxetine and venlaflaxine) and are commonly referred to as ‘dual-action’ drugs. However, it remains unclear exactly how antidepressants work in patients with major depressive disorder [23, 24]. The ‘monoamine hypothesis’ proposes that diminished activity of serotonergic, noradrenergic, and dopaminergic pathways plays a causal role in the pathophysiology of depression [25–27], but the role of serotonin in the pathophysiology and treatment of major depressive disorder is still unclear due to unreliable clinical biochemical findings and the difficulty of relating changes in serotonin activity to mood state [28].

### Why is it important to do this review?

It has been repeatedly shown that antidepressants seem to reduce depressive symptoms with a statistically significant effect, but the effect sizes are small or minimal and without importance to patients [10, 29, 30]. A recent review of both within patient and between patient anchor-based approaches suggested that the minimal clinically important difference on the Hamilton Depression Rating Scale 17 (HDRS-17) is likely to be in the range from 3 to 5 points [31]. Furthermore, there is inconsistent evidence concerning individual variability in who benefits from antidepressants [32, 33]. Considering this inconsistency, one must still assume that the average effect of antidepressants applies also to the individual patient [33, 34]. In addition, quality of life has previously been selectively reported in placebo-controlled trials of antidepressants [35]. Therefore, the beneficial effects of antidepressants are currently unclear.

When establishing evidence for any intervention, the beneficial *and* harmful effects must be carefully assessed [36]. If benefits are small or unimportant, society has less tolerability of risks of adverse events. Harmful effects are often insufficiently reported in journal articles compared to trial registries, causing significant under-reporting of harms associated with antidepressants [37, 38]. This might be the cause for conflicting evidence on whether antidepressants may trigger harmful effects in adults with major depressive disorder [39].

A meta-analysis was published in BMJ in 2009 [40] assessing the risk of suicidality in randomised clinical trials of antidepressants based on proprietary data submitted to US Food and Drug Administration (FDA). The meta-analysis included major depressive disorder, other depression, other psychiatric disorders, and non-psychiatric disorders. The authors concluded that risk of suicidality associated with use of antidepressants is strongly age dependent [40]. For suicidal behaviour or ideation and for suicidal behaviour only, the respective odds ratios were 1.62 (95% confidence interval [CI] 0.97 to 2.71) and 2.30 (95% CI 1.04 to 5.09) for participants aged < 25 years, 0.79 (95% CI 0.64 to 0.98) and 0.87 (95% CI 0.58 to 1.29) for those aged 25 to 64 years, and 0.37 (95% CI 0.18 to 0.76) and 0.06 (95% CI 0.01 to 0.58) for those aged ≥ 65 years. However, these age group subgroup analyses were not predefined in a registered or published protocol and should therefore be interpreted with caution.

In a study by Khan et al. [41], the Integrated Safety Summary data from approval packets for 14 investigational antidepressant programmes (1991–2013, 40,857 patients, 10,890 exposure years) were used to calculate suicides and suicide attempts per 100,000 patient exposure years for antidepressant and placebo treatment groups separately in patients with major depressive disorder. The study concluded that deaths by suicide and suicide attempts had decreased significantly in clinical trials assessing the effect of antidepressants following the year 2000 compared to the decade before 2000, and assessments of drug-placebo differences in suicide and suicide attempt rates revealed no significant differences [41]. However, a reanalysis of the data from this study found different results [39]. According to the reanalysis, there were 37 suicides (0.116%) and 206 suicide attempts (0.713%) in the antidepressant group versus 4 suicides (0.040%) and 28 suicide attempts (0.300%) in the placebo group. Thus, the suicide rate was significantly higher in the antidepressant group than in the placebo group (odds ratio [OR] 2.83; 95% CI 1.13 to 9.67, *p* = 0.02).

A large network meta-analysis was published in *The Lancet* in 2019 [42]. The authors included placebo-controlled and head-to-head trials of 21 commonly used antidepressants [42]. The authors recorded all outcomes as close to eight weeks as possible, that is, only short-term results were assessed. In this study, neither serious nor non-serious adverse events were assessed. Instead, the authors assessed ‘acceptability’ (treatment discontinuation measured by the proportion of participants who withdrew for any reason) and the proportion of participants who dropped out early because of adverse effects. But these outcomes are difficult to interpret clinically; participants might, for example, continue taking antidepressants even if they experience serious adverse effects.

We previously published a systematic review assessing the effects of the most commonly used antidepressants, SSRIs [29]. This review assessed both beneficial and harmful effects of SSRIs. The results showed that there was a significant effect of SSRIs on depressive symptoms, but the effect was of questionable clinical relevance and comparable to that of the network meta-analysis [42]. Moreover, we found almost no data on suicidal behaviour, and SSRIs significantly increased the risk of both serious and non-serious adverse events [29].

No former review has systematically assessed the beneficial and harmful effects of antidepressants including all types of antidepressants including both published trials and unpublished data from clinical study reports. Therefore, there is an urgent need for such a review. The present systematic review aims at forming the basis for evidence-based guideline recommendations for the use of antidepressants for major depressive disorder taking bias risks (systematic errors), play of chance (random errors), and certainty of the findings into consideration.

## Methods

The present protocol has been registered in the PROSPERO database (CRD42020220279) and is reported in accordance with the reporting guidance provided in the Preferred Reporting Items for Systematic Reviews and Meta-Analysis Protocols (PRISMA-P) statement [43, 44] (see checklist in Additional file 1).

### Criteria for considering studies for this review

#### Types of studies

We will include randomised clinical trials irrespective of setting, publication status, publication year, and language. We will not include quasi-randomised trials, cluster-randomised trials, or observational studies.

#### Types of participants

Adults (as defined by trialists) with a primary diagnosis of major depressive disorder as defined by standardised diagnostic criteria from either DSM-5 [6], ICD-11 [7], or earlier versions of these diagnostic manuals. Participants will be included irrespective of sex and comorbidities.

#### Types of interventions

As experimental intervention, we will accept the following: agomelatine, amineptine, amitriptyline, bupropion, butriptyline, cianopramine, citalopram, clomipramine, dapoxetine, demexiptiline, desipramine, desvenlafaxine, dibenzepin, dosulepin, dothiepin, doxepin, duloxetine, escitalopram, fluoxetine, fluvoxamine, imipramine, iprindole, levomilnacipran, lofepramine, maprotiline, melitracen, metapramine, milnacipran, mirtazapine, nefazodone, nortriptyline, noxiptiline, opipramol, paroxetine, protriptyline, quinupramine, reboxetine, sertraline, trazodone, tianeptine, trimipramine, venlafaxine, vilazodone, and vortioxetine. We will accept any of these antidepressants as experimental interventions irrespective of dose and duration of administration.

As control intervention, we will accept the following: placebo, ‘active placebo’ (a matching placebo that produces noticeable adverse effects that may convince the participant being treated and the blinded outcome assessors that the participants are receiving an active intervention), or no intervention. We will accept any of these control interventions irrespective of dose and duration of administration.

#### Cointerventions

We will accept any cointervention, if the cointervention is planned to be delivered similarly in the intervention and control groups.

### Outcome measures

#### Primary outcomes

1. Depressive symptoms measured on the 17-item or 21-item Hamilton Depression Rating Scale (HDRS) [45]
2. Proportion of participants with one or more serious adverse events. We will use the International Conference on Harmonisation of technical requirements for registration of pharmaceuticals for human use—Good Clinical Practice (ICH-GCP) definition of a serious adverse event, which is any untoward medical occurrence that resulted in death, was life-threatening, required hospitalisation or prolonging of existing hospitalisation, and resulted in persistent or significant disability or jeopardised the participant [46]. If the trialists do not use the ICH-GCP definition, we will include the data if the trialists use the term ‘serious adverse event.’ If the trialists do not use the ICH-GCP definition nor use the term serious adverse event, then we will also include the data, if the event clearly fulfils the ICH-GCP definition for a serious adverse event. We will secondly assess each serious adverse event separately.
3. Quality of life

#### Secondary outcomes

1. Proportion of participants with either a suicide or a suicide attempt (as defined by the trialists)
2. Suicidal ideation
3. Proportion of participants with one or more non-serious adverse events (any adverse event not classified as serious). We will secondly assess each non-serious adverse event separately.

#### Exploratory outcomes

1. Depressive symptoms measured on the Montgomery-Asberg Depression Rating Scale (MADRS) [47], the Beck’s Depression Inventory (BDI) [48], or the 6-item HDRS [49].
2. Proportion of participants in remission (as defined by trialists)
3. Proportion of participants achieving response (as defined by trialists)

#### Assessment time points

We will assess all our outcomes at maximum follow-up.

### Search methods for identification of studies

#### Electronic searches

We will search Cochrane Central Register of Controlled Trials (CENTRAL) in the Cochrane Library, MEDLINE Ovid, Embase Ovid, Latin American and Caribbean Health Sciences Literature (LILACS; Bireme), PsycINFO (EBSCO host), Science Citation Index Expanded (SCI-EXPANDED; Web of Science), Conference Proceedings Citation Index—Science (CPCI-S; Web of Science), Social Sciences Citation Index (SSCI; Web of Science), Conference Proceedings Citation Index—Social Science & Humanities (CPCI-SSH; Web of Science), Chinese Biomedical Literature Database (CBM), China Network Knowledge Information (CNKI), Chinese Science Journal Database (VIP), and Wafang Database to identify relevant trials. We will search all databases from their inception to the present. For a detailed search strategy for all electronic databases, see Additional file 2. The search strategies for the Chinese databases will be given at review stage. Trials will be included irrespective of language, publication status, publication year, and publication type.

#### Searching other resources

We will include the data from a recent systematic review on 21 antidepressants by Cipriani et al. [42]. The authors of this comprehensive review made the data available in a public repository. This is the largest database of new-generation antidepressants for the acute treatment of major depressive disorder compiled so far. Further, the reference lists of relevant publications will be checked for any unidentified randomised trials. We will contact the authors of included trials by email asking for unpublished randomised trials. To identify unpublished trials, we will also search clinical trial registers, websites of pharmaceutical companies, websites of US Food and Drug Administration (FDA), and European Medicines Agency (EMA). We will request FDA, EMA, and national medicines agencies to provide all publicly releasable information about relevant randomised clinical trials of antidepressants that were submitted for marketing approval, including clinical study reports [50]. Additionally, we will hand search conference abstracts from psychiatry conferences for relevant trials. We will also include unpublished and grey literature trials if we identify these and assess relevant retraction statements and errata for included trials.

### Data collection and analysis

We will perform and report the review following the recommendations stated in the Cochrane Handbook for Systematic Reviews of Interventions [36]. Analyses will be performed using Stata version 16.1 (StataCorp LLC, College Station, TX, USA) [51] and Trial Sequential Analysis [52, 53].

#### Selection of studies

Two review authors will independently screen titles and abstracts. We will retrieve all relevant full-text study reports/publications, and two review authors will independently screen the full text and identify and record reasons for exclusion of the ineligible studies. The two review authors will resolve any disagreement through discussion, or, if required, they will consult a third author.

#### Data extraction and management

Two authors will independently extract data from included trials in a dedicated data extraction sheet developed for this review. Disagreements will be resolved by discussion with a third author. The two review authors will assess duplicate publications and companion papers of a trial together to evaluate all available data simultaneously (maximise data extraction, correct bias assessment). We will contact the trial authors by email to specify any additional data, which may not have been reported sufficiently or at all in the publication.

#### Trial characteristics

We will extract the following data: bias risk components (as defined below), trial design (parallel, factorial, or crossover), number of intervention groups, length of follow-up, estimation of sample size, and inclusion and exclusion criteria.

#### Participant characteristics

We will extract the following data: number of randomised participants, number of analysed participants, number of participants lost to follow-up/withdrawals/crossover, age range (mean or median), and sex ratio.

#### Intervention characteristics

We will extract the following data: type of antidepressant, dose of intervention, and duration of intervention.

#### Control characteristics

We will extract the following data: type of control intervention, dose of intervention, and duration of intervention.

#### Outcomes

All outcomes listed above will be extracted from each randomised clinical trial, and we will identify if outcomes are incomplete or selectively reported according to the criteria described later in ‘incomplete outcome data’ bias domain and ‘selective outcome reporting’ bias domain.

### Notes

We will search for information regarding industry funding of either personal or academic activities for each trial author. We will judge a publication at high risk of for-profit bias if a trial is sponsored by the industry or if just one author has affiliation to the industry. We will note in the ‘Characteristics of included studies’ table, if outcome data were not reported in a usable way. Two review authors will independently transfer data into the Stata file [51]. Disagreements will be resolved through discussion, or if required, we will consult with a third author.

### Assessment of risk of bias in the included trials

Our bias risk assessment will be based on the Cochrane Risk of Bias tool-version 2 (RoB 2) as recommended in The Cochrane Handbook of Systematic Reviews of Interventions [36]. We will evaluate the methodology in respect of the following bias domains:

### Bias arising from the randomisation process

- *Low risk of bias.* Allocation was adequately concealed, AND there are no baseline imbalances across intervention groups at baseline appear to be compatible with chance, AND an adequate (random or otherwise unpredictable) method was used to generate allocation sequence, OR there is no information about the method used to generate the allocation sequence.
- *Some concerns.* Allocation was adequately concealed, AND there is a problem with the method of sequence generation, OR baseline imbalances suggest a problem with the randomisation process, OR no information is provided about concealment of allocation, AND baseline imbalances across intervention groups appear to be compatible with chance, OR no information to answer any of the signalling questions,
- *High risk of bias.* Allocation sequence was not concealed, OR no information is provided about concealment of allocation sequence, AND baseline imbalances suggest a problem with the randomisation process.

### Bias due to deviation from intended interventions

- *Low risk of bias.* Participants, carers, and personnel were unaware of intervention groups during the trial, OR participants, carers, or personnel were aware of intervention groups during the trial but any deviations from intended intervention reflected usual practice, OR participants, carers, or personnel were aware of intervention groups during the trial but any deviations from intended intervention were unlikely to impact on the outcome, AND no participants were analysed in the wrong intervention groups (that is, on the basis of intervention actually received rather than of randomised allocation).
- *Some concerns.* Participants, carers, or personnel were aware of intervention groups and there is no information on whether there were deviations from usual practice that were likely to impact on the outcome and were imbalanced between intervention groups, OR some participants were analysed in the wrong intervention groups (on the basis of intervention actually received rather than of randomised allocation) but there was little potential for a substantial impact on the estimated effect of intervention.
- *High risk of bias.* Participants, carers, or personnel were aware of intervention groups, and there were deviations from intended interventions that were unbalanced between the intervention groups and likely to have affected the outcome, OR some participants were analysed in the wrong intervention groups (on the basis of intervention actually received rather than of randomised allocation), and there was potential for a substantial impact on the estimated effect of intervention.

### Bias due to missing outcome data

- *Low risk of bias.* No missing data OR non-differential missing data (similar proportion of and similar reasons for missing data in compared groups) OR evidence of robustness of effect estimate to missing data (based on adequate statistical methods for handling missing data and sensitivity analysis)
- *Some concerns.* An unclear degree of missing data or unclear information on proportion and reasons for missingness in compared groups AND there is no evidence that the effect estimate is robust to missing data
- *High risk of bias.* A high degree of missing data AND differential missing data (different proportion of or different reasons for missing data in compared groups) AND there is no evidence that the effect estimate is robust to missing data

### Bias in measurement of outcomes

- *Low risk of bias.* The outcome assessors were unaware of the intervention received by study participants, OR the outcome assessors were aware of the intervention received by study participants, but the assessment of the outcome was unlikely to be influenced by knowledge of the intervention received.
- *Some concerns.* There is no information available to determine whether the assessment of the outcome is likely to be influenced by knowledge of the intervention received.
- *High risk of bias.* The assessment of the outcome was likely to be influenced by knowledge of the intervention received by study participants.

### Bias arising from selective reporting of results

- *Low risk of bias.* Reported outcome data are unlikely to have been selected, on the basis of the results, from multiple outcome measurements (e.g. scales, definitions, time points) within the outcome domain, and reported outcome data are unlikely to have been selected, on the basis of the results, from multiple analyses of the data.
- *Some concerns*. There is insufficient information available to exclude the possibility that reported outcome data were selected, on the basis of the results, from multiple outcome measurements (e.g. scales, definitions, time points) within the outcome domain, or from multiple analyses of the data. Given that analysis intentions are often unavailable or not reported with sufficient detail, we anticipate that this will be the default judgement for most trials.
- *High risk of bias*. Reported outcome data are likely to have been selected, on the basis of the results, from multiple outcome measurements (e.g. scales, definitions, time points) within the outcome domain, or from multiple analyses of the data (or both).

### Overall assessment of risk of bias

- *Low risk of bias*. The trial is judged to be at low risk of bias for all domains.
- *High risk of bias*. The trial is judged to be at high risk of bias or to be at some concerns in at least one domain. Our subgroup analysis will compare the intervention effect of trials at low risk of bias with trials at high risk of bias, that is one or more domains at some concern or high risk of bias.

We will assess the domains ‘missing outcome data’, ‘risk of bias in measurement of the outcome’, and ‘risk of bias in selection of the reported result’ for each outcome result. Thus, we can assess the bias risk for each outcome assessed in addition to each trial. Our primary conclusions will be based on the results of our primary outcome results with overall low risk of bias. Both our primary and secondary conclusions will be presented in the ‘Summary of findings’ tables.

#### Differences between the protocol and the review

We will conduct the review according to this published protocol and report any deviations from it in the ‘Differences between the protocol and the review’ section of the systematic review.

#### Measurement of treatment effect

##### Dichotomous outcomes

We will calculate risk ratios (RRs) with 95% confidence interval (CI) for dichotomous outcomes, as well as the Trial Sequential Analysis-adjusted CIs (see the following).

##### Continuous outcomes

We will calculate the mean differences (MDs) and consider calculating the standardised mean difference (SMD) with 95% CI for continuous outcomes. We will also calculate Trial Sequential Analysis-adjusted CIs (see the following).

#### Dealing with missing data

We will use intention-to-treat data if provided by the trialists [54]. We will, as the first option, contact all trial authors to obtain any relevant missing data (i.e. for data extraction and for assessment of risk of bias, as specified above).

##### Dichotomous outcomes

We will not impute missing values for any outcomes in our primary analysis. In our sensitivity analyses (see the following paragraph), we will impute data.

##### Continuous outcomes

We will primarily analyse scores assessed at single time points. If only changes from baseline scores are reported, we will analyse the results together with follow-up scores [36]. If standard deviations (SDs) are not reported, we will calculate the SDs using trial data, if possible. We will not use intention-to-treat data if the original report did not contain such data. We will not impute missing values for any outcomes in our primary analysis. In our sensitivity analysis (see the following paragraph) for continuous outcomes, we will impute data.

#### Assessment of heterogeneity

We will primarily investigate forest plots to visually assess any sign of heterogeneity. We will secondly assess the presence of statistical heterogeneity by chi^2^ test (threshold P < 0.10) and measure the quantities of heterogeneity by the I^2^ statistic [55, 56]. We will investigate possible heterogeneity through subgroup analyses. We may ultimately decide that a meta-analysis should be avoided [36].

#### Assessment of reporting biases

We will use a funnel plot to assess reporting bias if ten or more trials are included. We will visually inspect funnel plots to assess the risk of bias. We are aware of the limitations of a funnel plot (i.e. a funnel plot assesses bias due to small sample size). From this information, we assess possible reporting bias. For dichotomous outcomes, we will test asymmetry with the Harbord test [57] if τ^2^ is less than 0.1 and with the Rücker test if τ^2^ is more than 0.1. For continuous outcomes, we will use the regression asymmetry test [58] and the adjusted rank correlation [59].

#### Unit of analysis issues

We will only include randomised clinical trials. For trials using crossover design, only data from the first period will be included [36, 60]. There will therefore not be any unit of analysis issues. We will not include cluster-randomised trials, due to their problems with randomisation, and blinding.

#### Data synthesis

##### Meta-analysis

We will undertake the meta-analysis according to The Cochrane Handbook for Systematic Reviews of Interventions [36], Keus et al. [61], and our eight-step procedure suggested by Jakobsen et al. [62]. We will use the statistical software Stata version 16 to analyse data [51]. We will assess our intervention effects with both random-effects model meta-analyses (Hartung-Knapp-Sidik-Jonkman) [63] and fixed-effect model meta-analyses (Mantel-Haenszel for dichotomous outcomes and inverse variance for continuous outcomes) [36, 64]. We will use the more conservative point estimate of the two [62]. The more conservative point estimate is the estimate with the highest p-value. We assess a total of six primary and secondary outcomes, and we will therefore consider a p-value of 0.014 or less as the threshold for statistical significance [62]. We will investigate possible heterogeneity through subgroup analyses. We will use our eight-step procedure to assess if the thresholds for significance are crossed [62]. This eight-step procedure comprise of the following steps: (1) obtain the 95% confidence intervals and the *P*-values from both fixed-effect and random-effects meta-analyses and report the most conservative results as the main results, (2) explore the reasons behind substantial statistical heterogeneity using subgroup and sensitivity analyses (see step 6), (3) to take account of problems with multiplicity adjust the thresholds for significance according to the number of primary outcomes (we will both adjust the thresholds for significance according to the number of primary and secondary outcomes), (4) calculate required information sizes (≈ the a priori required number of participants for a meta-analysis to be conclusive) for all outcomes and analyse each outcome with trial sequential analysis. Report whether the trial sequential monitoring boundaries for benefit, harm, or futility are crossed, (5) calculate Bayes factors for all primary outcomes, (6) use subgroup analyses and sensitivity analyses to assess the potential impact of bias on the review results, (7) assess the risk of publication bias, and (8) assess the clinical significance of the statistically significant review results [62].

Where multiple trial arms are reported in a single trial, we will include only the relevant arms. If two comparisons are combined in the same meta-analysis, we will halve the control group (participants and amount of evens to avoid double-counting). For continuous data, we will keep the main score [36]. Trials with a factorial design will be included. In case of, e.g. a 2 × 2 factorial designed trial, the two groups receiving antidepressants will be considered experimental groups, while the two groups receiving placebo, ‘active placebo’, or no intervention will be considered control groups.

##### Trial Sequential Analysis

Traditional meta-analysis runs the risk of random errors due to sparse data and repetitive testing of accumulating data when updating reviews. We wish to control the risks of type I errors and type II errors. We will therefore perform Trial Sequential Analysis on all outcomes, in order to calculate the diversity-adjusted required information size (DARIS; that is, the number of participants needed in a meta-analysis to detect or reject a certain intervention effect) and the cumulative Z-curve’s breach of relevant trial sequential monitoring boundaries [52, 53, 65–71]. A more detailed description of Trial Sequential Analysis software can be found in the manual [53] and at http://www.ctu.dk/tsa/. For dichotomous outcomes, we will estimate the required information size based on the observed proportion of patients with an outcome in the control group (the cumulative proportion of patients with an event in the control groups relative to all patients in the control groups), a relative risk reduction or a relative risk increase of 20%, an alpha of 1.6% for all our outcomes, a beta of 10%, and the observed diversity as suggested by the trials in the meta-analysis. For continuous outcomes, we will in the Trial Sequential Analysis use the observed standard deviation (SD) in the control group, a mean difference of three HDRS points when assessing depressive symptoms (for other continuous outcomes the observed SD/2), an alpha of 1.6% for all outcomes, a beta of 10%, and the observed diversity as suggested by the trials in the meta-analysis.

#### Subgroup analysis and integration of heterogeneity

##### Subgroup analysis

We will perform the following subgroup analyses when analysing the primary outcomes (depressive symptoms, serious adverse events, quality of life).

1. Trials at high risk of bias compared to trials at low risk of bias
2. Trials with for-profit bias compared to trials at unknown or known risk of for-profit bias [72]
3. Types of antidepressant drug
4. Types of comparator (placebo, ‘active placebo’, no intervention)
5. Age groups (18 to 24 years, 25 to 64 years, ≥ 65 years)

We will use the formal test for subgroup interactions in Stata [51].

##### Sensitivity analysis

To assess the potential impact of the missing data for dichotomous outcomes, we will perform the two following sensitivity analyses on both the primary and secondary dichotomous outcomes.

- *‘Best-worst-case’ scenario*. We will assume that all participants lost to follow-up in the antidepressant group survived, had no serious adverse events, had no suicides or suicide attempts, and had no non-serious adverse events, and that all those participants lost to follow-up in the control group did not survive, had a serious adverse event, died by suicide or had a suicide attempt, and had a non-serious adverse event.
- *‘Worst-best-case’ scenario.* We will assume that all participants lost to follow-up in the antidepressant group did not survive, had a serious adverse event, died by suicide or had a suicide attempt, and had a non-serious adverse event, and that all those participants lost to follow-up in the control group survived, had no serious adverse events, had no suicides or suicide attempts, and had no non-serious adverse events.

We will present results of both scenarios in our review. When analysing depressive symptoms, suicidal ideation, and quality of life, a ‘beneficial outcome’ will be the group mean plus two SDs (we will secondly use one SD in another sensitivity analysis) of the group mean and a ‘harmful outcome’ will be the group mean minus two SDs (we will secondly use one SD in another sensitivity analysis) of the group mean [62]. To assess the potential impact of missing SDs for continuous outcomes, we will perform the following sensitivity analysis:

- Where SDs are missing and it is not possible to calculate them, we will impute SDs from trials with similar populations and low risk of bias. If we find no such trials, we will impute SDs from trials with a similar population. As the final option, we will impute the mean SD from all included trials.

We will present results of this scenario in our review. Other post hoc sensitivity analyses might be warranted if unexpected clinical or statistical heterogeneity is identified during the analysis of the review results [62].

##### ‘Summary of findings’ tables

We will create summary of findings tables for each comparison including each of the prespecified primary and secondary outcomes (depressive symptoms, serious adverse events, quality of life, suicide or suicide attempt, non-serious adverse events). We will use the five Grading Recommendations Assessment Development Evaluation (GRADE) considerations (bias risk, heterogeneity, imprecision, indirectness, and publication bias) to assess the certainty of evidence [62, 73–75]. We will assess imprecision using Trial Sequential Analysis. We will downgrade imprecision in GRADE by two levels if the accrued number of participants is below 50% of the DARIS, and one level if between 50 and 100% of DARIS. We will not downgrade if the cumulative Z-curve crosses the monitoring boundaries for benefit, harm, or futility, or if DARIS is reached. We will justify all decisions to downgrade the quality of evidence using footnotes, and we will make comments to aid the reader’s understanding of the assessment where necessary. Firstly, we will present our results in the summary of findings tables based on the results from the trials with overall low risk of bias, and secondly, we will present the results based on all trials.

### Project plan

We will publish separate systematic reviews assessing the beneficial and harmful effects of the most frequently used antidepressants. We will subsequently gather data from all these reviews, update the searches and analyses, and finally publish the overall results from all antidepressants in a large publication. We will publish the following protocols and systematic reviews separately: (1) tricyclic antidepressants, (2) SSRIs, (3) venlaflaxine, (4) mirtazapine, and (5) duloxetine.

## Discussion

This protocol aims at assessing the beneficial and harmful effects of antidepressants versus placebo, ‘active placebo’, or no intervention in adults with major depressive disorder. Primary outcomes will be depressive symptoms, serious adverse events, and quality of life. Secondary outcomes will be suicide or suicide attempts, suicidal ideation, and non-serious adverse events.

Our protocol has a number of strengths. The predefined methodology is based on Cochrane methodology [36], PRISMA [76, 77], Keus et al. [61], our eight-step assessment suggested by Jakobsen et al. [62], Trial Sequential Analysis [52], and GRADE assessment [73–75]. Hence, this protocol considers both risks of random errors and risks of systematic errors [62]. Further, we increase the statistical power by pooling various antidepressants as the experimental intervention. Moreover, we will include both unpublished and published trials as well as clinical study reports [50].

Our protocol also has limitations. The primary limitation is the potential for high statistical heterogeneity as a result of including various antidepressants as the experimental intervention. To minimise this limitation, we will carefully look for signs of heterogeneity and ultimately decide if data ought to be pooled and meta-analysed, and we have planned several subgroup analyses. Another limitation is the large number of analyses which increases the risk of type 1 error. We have adjusted our thresholds for significance according to the number of primary and secondary outcomes, but we have not adjusted our thresholds for significance according to the total number of comparisons (e.g. subgroup analyses and sensitivity analyses). As mentioned in the ‘Background’ section, we expect inadequate reporting of harmful effects in the included trials, which increases the risk of underestimation of harmful effects. Finally, we expect short follow-up periods.

## Supplementary Information

**Additional file 1.** PRISMA-P (Preferred Reporting Items for Systematic review and Meta-Analysis Protocols) 2015 checklist.

**Additional file 2.** Search strategies.

## Data Availability

Data sharing is not applicable to this protocol article.

## Abbreviations

BDI: Beck’s Depression Inventory
CBM: Chinese Biomedical Literature Database
CENTRAL: Cochrane Central Register of Controlled Trials
CI: Confidence interval
CNKI: China Network Knowledge Information
CPCH-SSH: Conference Proceedings Citation Index—Social Science & Humanities
CPCI-S: Conference Proceedings Citation Index—Science
DARIS: diversity-adjusted required information size
EMA: European Medicines Agency
FDA: US Food and Drug Administration
GRADE: Grading of Recommendations Assessment, Development and Evaluation
HDRS: Hamilton Depression Rating Scale
ICH-GCP: Good Clinical Practice
LILACS: Latin American and Caribbean Health Sciences Literature
MADRS: Montgomery-Asberg Depression Rating Scale
MAOIs: Monoamine oxidase inhibitors
MDs: Mean differences
NICE: UK National Institute for Health and Care Excellence
Prisma: Preferred Reporting Items for Systematic Reviews and Meta-Analysis Protocols
ROB2: Cochrane Risk of Bias tool-version 2
RRs: Risk ratios
SCI-EXPANDED: Science Citation Index Expanded
SDs: Standard deviations
SMD: Standardised mean difference
SNRIs: Serotonin and norepinephrine reuptake inhibitors
SSCI: Social Sciences Citation Index
SSRIs: Selective serotonin reuptake inhibitors
TCA: Tricyclic antidepressants
VIP: Chinese Science Journal Database
WHO: World Health Organization
