# Supplementary material for: Beneficial and harmful effects of antidepressants versus placebo, ‘active placebo’, or no intervention for adults with major depressive disorder: a protocol for a systematic review of published and unpublished data with meta-analyses and trial sequential analyses
Source: Syst Rev. 2021 May 25;10:154. doi: 10.1186/s13643-021-01705-6 (PMC8152051; doi:10.1186/s13643-021-01705-6)
Supplement: Supplementary file 2 — Additional file 2. Search strategies. [file 13643_2021_1705_MOESM2_ESM.doc]

# Search strategies for

# ‘Antidepressants for major depressive disorder’

**(S Juul)**

Cochrane Central Register of Controlled Trials (CENTRAL) in the Cochrane Library (Latest issue)

#1 MeSH descriptor: [Antidepressive Agents] explode all trees

#2 (antidepress* or (moodstimula* or mood-stimula*) or thymoanaleptic* or thymoleptic*)

#3 (Agomelatine or Amineptine or Amitriptyline or Ariclaim or Aropax or Avanza or Bupropion or Butriptyline or Celexa or Cipra* or Citalopram or Clomipramine or Combar or Cymbalta or Dapoxetine or Desipramine or Desvenlafaxine or Dibenzepin or Dosulepin or Doxepin or Duloxetine or Dumirox or Duzela or Ef*exor or Escitalopram or Esertia or Fevarin or Fluoxetine or Fluvoxamine or Fontex or Imipramine or Iprindole or Levomilnacipran or Lexam* or Lexapro or Lofepramine or Lustral or Luvox or Maprotiline or Milnacipran or Miro or Mirtazapine or Nefazodone or Nortriptyline or Opipramol or Paroxetine or Paxil or Pexeva or Protriptyline or Prozac or Rapiflux or Reboxetine or Remeron or Selfemra or Serafem or Sereupin or Seroplex or Seroxat or Sertraline or Tianeptine or Trazodone or Trimipramine or Venlafaxine or Vilazodone or Vortioxetine or Xeristar or Yentreve or Zispin or Zoloft)

#4 #1 or #2 or #3

#5 MeSH descriptor: [Depressive Disorder, Major] explode all trees

#6 MeSH descriptor: [Depressive Disorder] this term only

#7 MeSH descriptor: [Seasonal Affective Disorder] explode all trees

#8 MeSH descriptor: [Dysthymic Disorder] explode all trees

#9 MeSH descriptor: [Depression] explode all trees

#10 MeSH descriptor: [Affective Symptoms] this term only

#11 ((depress* or affective or dysthym*) and (disorder* or disease* or symptom*))

#12 #5 or #6 or #7 or #8 or #9 or #10 or #11

#13 #4 and #12

MEDLINE Ovid (1946 to present)

1. exp Antidepressive Agents/

2. (antidepress* or (moodstimula* or mood-stimula*) or thymoanaleptic* or thymoleptic*).mp. [mp=title, abstract, original title, name of substance word, subject heading word, floating sub-heading word, keyword heading word, organism supplementary concept word, protocol supplementary concept word, rare disease supplementary concept word, unique identifier, synonyms]

3. (Agomelatine or Amineptine or Amitriptyline or Ariclaim or Aropax or Avanza or Bupropion or Butriptyline or Celexa or Cipra* or Citalopram or Clomipramine or Combar or Cymbalta or Dapoxetine or Desipramine or Desvenlafaxine or Dibenzepin or Dosulepin or Doxepin or Duloxetine or Dumirox or Duzela or Ef*exor or Escitalopram or Esertia or Fevarin or Fluoxetine or Fluvoxamine or Fontex or Imipramine or Iprindole or Levomilnacipran or Lexam* or Lexapro or Lofepramine or Lustral or Luvox or Maprotiline or Milnacipran or Miro or Mirtazapine or Nefazodone or Nortriptyline or Opipramol or Paroxetine or Paxil or Pexeva or Protriptyline or Prozac or Rapiflux or Reboxetine or Remeron or Selfemra or Serafem or Sereupin or Seroplex or Seroxat or Sertraline or Tianeptine or Trazodone or Trimipramine or Venlafaxine or Vilazodone or Vortioxetine or Xeristar or Yentreve or Zispin or Zoloft).mp. [mp=title, abstract, original title, name of substance word, subject heading word, floating sub-heading word, keyword heading word, organism supplementary concept word, protocol supplementary concept word, rare disease supplementary concept word, unique identifier, synonyms]

4. 1 or 2 or 3

5. exp Depressive Disorder, Major/

6. Depressive Disorder/

7. exp Seasonal Affective Disorder/

8. exp Dysthymic Disorder/

9. exp Depression/

10. Affective Symptoms/

11. ((depress* or affective or dysthym*) and (disorder* or disease* or symptom*)).mp. [mp=title, abstract, original title, name of substance word, subject heading word, floating sub-heading word, keyword heading word, organism supplementary concept word, protocol supplementary concept word, rare disease supplementary concept word, unique identifier, synonyms]

12. 5 or 6 or 7 or 8 or 9 or 10 or 11

13. 4 and 12

14. (randomized controlled trial or controlled clinical trial).pt. or clinical trials as topic.sh. or trial.ti.

15. (random* or blind* or placebo* or meta-analys*).mp. [mp=title, abstract, original title, name of substance word, subject heading word, floating sub-heading word, keyword heading word, organism supplementary concept word, protocol supplementary concept word, rare disease supplementary concept word, unique identifier, synonyms]

16. 13 and (14 or 15)

17. limit 16 to ("all adult (19 plus years)" or "adolescent (13 to 18 years)" or "young adult (19 to 24 years)" or "adult (19 to 44 years)" or "young adult and adult (19-24 and 19-44)" or "middle age (45 to 64 years)" or "middle aged (45 plus years)" or "all aged (65 and over)" or "aged (80 and over)")

Embase Ovid (1974 to present)

1. exp antidepressant agent/

2. (antidepress* or (moodstimula* or mood-stimula*) or thymoanaleptic* or thymoleptic*).mp. [mp=title, abstract, heading word, drug trade name, original title, device manufacturer, drug manufacturer, device trade name, keyword, floating subheading word, candidate term word]

3. (Agomelatine or Amineptine or Amitriptyline or Ariclaim or Aropax or Avanza or Bupropion or Butriptyline or Celexa or Cipra* or Citalopram or Clomipramine or Combar or Cymbalta or Dapoxetine or Desipramine or Desvenlafaxine or Dibenzepin or Dosulepin or Doxepin or Duloxetine or Dumirox or Duzela or Ef*exor or Escitalopram or Esertia or Fevarin or Fluoxetine or Fluvoxamine or Fontex or Imipramine or Iprindole or Levomilnacipran or Lexam* or Lexapro or Lofepramine or Lustral or Luvox or Maprotiline or Milnacipran or Miro or Mirtazapine or Nefazodone or Nortriptyline or Opipramol or Paroxetine or Paxil or Pexeva or Protriptyline or Prozac or Rapiflux or Reboxetine or Remeron or Selfemra or Serafem or Sereupin or Seroplex or Seroxat or Sertraline or Tianeptine or Trazodone or Trimipramine or Venlafaxine or Vilazodone or Vortioxetine or Xeristar or Yentreve or Zispin or Zoloft).mp. [mp=title, abstract, heading word, drug trade name, original title, device manufacturer, drug manufacturer, device trade name, keyword, floating subheading word, candidate term word]

4. 1 or 2 or 3

5. exp major depression/

6. depression/

7. exp seasonal affective disorder/

8. exp dysthymia/

9. emotional disorder/

10. ((depress* or affective or dysthym*) and (disorder* or disease* or symptom*)).mp. [mp=title, abstract, heading word, drug trade name, original title, device manufacturer, drug manufacturer, device trade name, keyword, floating subheading word, candidate term word]

11. 5 or 6 or 7 or 8 or 9 or 10

12. 4 and 11

13. Randomized controlled trial/ or Controlled clinical study/ or trial.ti.

14. (random* or blind* or placebo* or meta-analys*).mp. [mp=title, abstract, heading word, drug trade name, original title, device manufacturer, drug manufacturer, device trade name, keyword, floating subheading word, candidate term word]

15. 12 and (13 or 14)

16. limit 15 to (adult <18 to 64 years> or aged <65+ years>)

**LILACS (Bireme; 1982 to present)**

(antidepress$ or (moodstimula$ or mood-stimula$) or thymoanaleptic$ or thymoleptic$) or (Agomelatine or Amineptine or Amitriptyline or Ariclaim or Aropax or Avanza or Bupropion or Butriptyline or Celexa or Cipra$ or Citalopram or Clomipramine or Combar or Cymbalta or Dapoxetine or Desipramine or Desvenlafaxine or Dibenzepin or Dosulepin or Doxepin or Duloxetine or Dumirox or Duzela or Ef$exor or Escitalopram or Esertia or Fevarin or Fluoxetine or Fluvoxamine or Fontex or Imipramine or Iprindole or Levomilnacipran or Lexam$ or Lexapro or Lofepramine or Lustral or Luvox or Maprotiline or Milnacipran or Miro or Mirtazapine or Nefazodone or Nortriptyline or Opipramol or Paroxetine or Paxil or Pexeva or Protriptyline or Prozac or Rapiflux or Reboxetine or Remeron or Selfemra or Serafem or Sereupin or Seroplex or Seroxat or Sertraline or Tianeptine or Trazodone or Trimipramine or Venlafaxine or Vilazodone or Vortioxetine or Xeristar or Yentreve or Zispin or Zoloft) [Words] and ((depress$ or affective or dysthym$) and (disorder$ or disease$ or symptom$)) [Words]

**PsycINFO (EBSCO host; 1806 to present)**

S17 S15 AND S16

S16 TI adult* or Elder* or older or Geriatri* or Senil* or Old Age* or Late Life or Aged OR AB adult* or Elder* or older or Geriatri* or Senil* or Old Age* or Late Life or Aged

S15 S13 AND S14

S14 TX ( (random* or blind* or placebo* or meta-analys*) ) OR TI trial*

S13 S4 AND S12

S12 S5 OR S6 OR S7 OR S8 OR S9 OR S10 OR S11

S11 TX ((depress* or affective or dysthym*) and (disorder* or disease* or symptom*))

S10 MA Affective Symptoms

S9 MA Depression

S8 MA Dysthymic Disorder

S7 MA Seasonal Affective Disorder

S6 MA Depressive Disorder Expanders

S5 MA Depressive Disorder, Major

S4 S1 OR S2 OR S3

S3 TX (Agomelatine or Amineptine or Amitriptyline or Ariclaim or Aropax or Avanza or Bupropion or Butriptyline or Celexa or Cipra* or Citalopram or Clomipramine or Combar or Cymbalta or Dapoxetine or Desipramine or Desvenlafaxine or Dibenzepin or Dosulepin or Doxepin or Duloxetine or Dumirox or Duzela or Ef*exor or Escitalopram or Esertia or Fevarin or Fluoxetine or Fluvoxamine or Fontex or Imipramine or Iprindole or Levomilnacipran or Lexam* or Lexapro or Lofepramine or Lustral or Luvox or Maprotiline or Milnacipran or Miro or Mirtazapine or Nefazodone or Nortriptyline or Opipramol or Paroxetine or Paxil or Pexeva or Protriptyline or Prozac or Rapiflux or Reboxetine or Remeron or Selfemra or Serafem or Sereupin or Seroplex or Seroxat or Sertraline or Tianeptine or Trazodone or Trimipramine or Venlafaxine or Vilazodone or Vortioxetine or Xeristar or Yentreve or Zispin or Zoloft)

S2 TX (antidepress* or (moodstimula* or mood-stimula*) or thymoanaleptic* or thymoleptic*)

S1 MA Antidepressive Agents

**Science Citation Index Expanded (Web of Science; 1900 to present);** **Conference Proceedings Citation Index – Science (Web of Science; 1990 to present); Social Sciences Citation Index (Web of Science; 1956 to present), and Conference Proceedings Citation Index- Social Science & Humanities (Web of Science; 1990 to present)**

#7 #6 AND #5

#6 TI=(random* or blind* or placebo* or meta-analys* or trial*) OR TS=(random* or blind* or placebo* or meta-analys*)

#5 #4 AND #3

#4 TS=((depress* or affective or dysthym*) and (disorder* or disease* or symptom*))

#3 #2 OR #1

#2 TS=(Agomelatine or Amineptine or Amitriptyline or Ariclaim or Aropax or Avanza or Bupropion or Butriptyline or Celexa or Cipra* or Citalopram or Clomipramine or Combar or Cymbalta or Dapoxetine or Desipramine or Desvenlafaxine or Dibenzepin or Dosulepin or Doxepin or Duloxetine or Dumirox or Duzela or Ef*exor or Escitalopram or Esertia or Fevarin or Fluoxetine or Fluvoxamine or Fontex or Imipramine or Iprindole or Levomilnacipran or Lexam* or Lexapro or Lofepramine or Lustral or Luvox or Maprotiline or Milnacipran or Miro or Mirtazapine or Nefazodone or Nortriptyline or Opipramol or Paroxetine or Paxil or Pexeva or Protriptyline or Prozac or Rapiflux or Reboxetine or Remeron or Selfemra or Serafem or Sereupin or Seroplex or Seroxat or Sertraline or Tianeptine or Trazodone or Trimipramine or Venlafaxine or Vilazodone or Vortioxetine or Xeristar or Yentreve or Zispin or Zoloft)

#1 TS=(antidepress* or (moodstimula* or mood-stimula*) or thymoanaleptic* or thymoleptic*)
